# A semi-empirical model of the energy balance closure in the surface layer

**DOI:** 10.1371/journal.pone.0209022

**Published:** 2018-12-12

**Authors:** Frederik De Roo, Sha Zhang, Sadiq Huq, Matthias Mauder

**Affiliations:** Karlsruhe Institute of Technology, Institute of Meteorology and Climate Research – Atmospheric Environmental Research, Garmisch-Partenkirchen, Germany; Oak Ridge National Laboratory, UNITED STATES

## Abstract

It has been hypothesized that the energy balance closure problem of single-tower eddy-covariance measurements is linked to large-scale turbulent transport. In order to shed light on this problem, we investigate the functional dependence of the normalized residual for the potential temperature and humidity conservation equations, i.e. the imbalance ratio for the fluxes of latent and sensible heat. We set up a suite of simulations consisting of cases with different stability and surface Bowen ratio. We employ a nesting approach in the lower part of the atmospheric boundary-layer to achieve higher spatial resolution near the surface. Our simulations reproduce earlier simulation results for the mixed layer and also mimic the saw-blade pattern of real flux measurements. Focusing on homogeneous terrain, we derive a parameterization for the spatially averaged flux imbalance ratios of latent and sensible heat in the surface layer. We also investigate how the remaining imbalance for a given point measurement is related to the local turbulence, by deriving a statistical model based on turbulence characteristics that are related to large-scale turbulence. The average imbalance ratio scales well with friction velocity, especially for sensible heat. For the latent heat flux, our results show that the Bowen ratio also influences the underestimation. Furthermore, in the surface layer the residual has a linear dependence on the absolute height divided by the boundary-layer height. Our parameterization allows us to deduce an expression for the residual in the energy budget for a particular measurement half hour, based on the measurement height and stability.

## Introduction

There is a continuous exchange of energy and matter between the Earth’s biosphere and atmosphere. Many fundamental ecological and atmospheric processes are governed by exchanges and interactions through the interface between those two Earth-system-compartments. For example, about 70–80% of the entire solar energy absorbed by the Earth is partitioned into various channels of heat transfer [1]. Greenhouse gases are taken up or released by microorganisms, plants, animals, humans and machines, and water vapour, the most important of all greenhouse gases, is evaporated into the air where it can form clouds and precipitation. Therefore, quantitative knowledge about the biosphere-atmosphere exchanges is essential to predict the evolution of the planet’s ecosystems, weather and climate.

The principal transport mechanisms of biosphere-atmosphere exchange are by turbulent motion. The fact that a complete theoretical description of turbulence is not available is recognized as one of the fundamental unsolved problems of physics (turbulence closure problem). As a practical approach, semi-empirical parameterizations of turbulent transport in the atmospheric boundary layer are needed, which are in the end always based on experimental data [2–5]. In consequence, the accuracy of all turbulent flow models depends on the quality of their closure parameterizations, and thus the underlying turbulence measurements. This applies to weather forecasts, climate predictions as well as estimates of air pollutants dispersion.

One way to examine the validity of turbulence measurements is by the first law of thermodynamics, or more specifically, the closure of the energy balance at the Earth’s surface. Measurements from eddy-covariance sites all over the world show that the sum of turbulent energy fluxes (e.g. convective heat) between the biosphere and the atmosphere generally underestimate the non-convective terms by 10% to 30% [6–9]. Hence, considering the law of energy conservation, it follows that even state-of-the-art measurements are generally fraught with a substantial closure problem.

Several studies on the surface energy balance closure problem indicate that some scales of atmospheric motion, particularly the larger ones in the meso-*γ* range [10], inherently cannot be captured by single point-measurement systems, at least under certain conditions [9,11,12]. Because these large-scale structures typically do not propagate with the mean wind, these missing scales can only be studied by means of spatially-resolving measurements, e.g. airborne systems [11,13] and lidars [14], or by means of large-eddy simulations (LES) doing virtual measurements in a controlled environment. The pioneering LES study of the energy balance closure problem by Kanda et al. [15] confirmed the hypothesis that large-scale atmospheric motion causes a flux underestimation. More precisely, the underestimation is caused by secondary circulations, which develop under convective conditions either as turbulent organized structures (TOS) or as thermally-induced meso-scale circulations (TMC) [16]. Accordingly, these large-scale phenomena that extend across the entire boundary-layer affect the near-surface eddy-covariance measurements locally in form of advection by the mean flow and horizontal flux divergence [12,17–21]. Recently, Gao et al. [22] showed that large eddies may both increase or decrease the turbulent fluxes. In boundary-layer turbulence research, the primary flow commonly denotes the random background turbulence [23]. Secondary circulations are those circulations of the boundary-layer scale, e.g. rolls, the abovementioned TOS and TMC, or local valley-mountain systems.

In order to predict this systematic error of eddy-covariance measurements without prior knowledge of all non-turbulent terms of the surface energy balance, two semi-empirical parameterizations have been proposed in the literature. One approach, proposed by Panin and Bernhofer [24], focuses on the role of surface heterogeneities in generating advective fluxes by advancing the concept of Panin et al. [25]. This parameterization proposes only one correction factor that describes the mean closure of a given measurement site as a function of the ratio between the effective roughness length and the dominant horizontal scale of landscape inhomogeneities in the surrounding area. In contrast, the approach of Huang et al. [26] is designed to parametrize the energy balance closure for 30-min fluxes, concentrating on the TOS developing in a homogeneously heated boundary layer. They use virtual tower measurements in LES to develop a parameterization scheme, building on the findings from earlier LES studies [15,27]. According to this approach, the magnitude of the underestimation 〈*I*〉 depends on friction velocity u_*_, the convective velocity scale w_*_, and the ratio between measurement height *z* and the boundary layer height *z*_*i*_,

$$\langle I\rangle =f_{1}(\frac{u_{*}}{w_{*}})f_{2}(\frac{z}{z_{i}}),$$ (1)

where *f*_1_ and *f*_2_ represent empirical functions that are determined based on LES data.

Both methods have merit in shedding light on the processes underlying the energy balance closure problem, but neither can be applied for practical purposes without further modification in order to correct eddy-covariance measurements [28]. The approach of Panin and Bernhofer [24] does not take into account the effect of thermal surface heterogeneity [16,29] and complex terrain [30], and it does not consider the effect of changing atmospheric stability [15,31]. The latter effect is included in the approach of Huang et al. [26], but it does not take into account any effects related to the surface heterogeneity of a specific site and, most of all, their correction function is not valid for measurement heights within the surface layer where eddy-covariance measurements are generally conducted.

Nevertheless, LES is an excellent tool for studying the energy balance closure problem because every relevant information is known everywhere in the model domain. However, one of the most critical limitations of previous LES studies has been the too coarse grid-resolution of the near-surface processes, especially since the domain also had to be sufficiently large in order to allow for the development of secondary circulations. Therefore, the energy balance residual always vanished towards the surface since the vertical grid spacing was only around 20 m and typically LES results are not fully reliable in the lowest 5 to 10 grid levels. Interestingly, a recently published LES study with 2 m vertical grid spacing [32] found that organized structures of the size of the boundary layer affect the turbulent transport at the canopy top and even within the canopy. These findings indirectly support the secondary circulation hypothesis for the energy balance closure problem. However, this aspect was not investigated quantitatively in that study [31].

Hence, the aim of our work is to conduct a parameter study of the energy balance closure problem using LES with sufficiently fine grid-spacing near the surface, so that typical heights of eddy-covariance towers are resolved. To facilitate this, we will employ a newly developed vertical grid-nesting method [33]. We will test the predictive power of different parameters, including the ones proposed by Huang et al. [26] but also others that have been suggested in the literature, e.g. the Bowen ratio Bo [30], the temperature and humidity difference between near surface air and a higher air layer [34], the correlation coefficient between the horizontal and vertical velocity *R*_*uw*_ [35], and also mixed third moments ($\overset{-}{w^{\prime }w^{\prime }T^{\prime }}$ and $\overset{-}{w^{\prime }w^{\prime }q^{\prime }}$), which can be connected to coherent structures [36].

## Methods

Our simulations were performed with the LES model PALM version 4.0 [37]. Additionally, a vertical nesting scheme was implemented between a coarse grid LES and a fine grid LES [33] to allow for finer resolution near the surface. PALM resolves the turbulence down to the scale of the grid spacing, any subgrid-scale turbulence is parameterized. The closure model in PALM is a 1.5-order closure scheme [38], where the equations for the resolved velocities and scalars are derived by implicit filtering over each grid box of the turbulent Navier-Stokes equations, and where an additional prognostic equation for the turbulent kinetic energy is introduced. The turbulent kinetic energy in PALM (the sum of the spatial variance of the subgrid-scale instantaneous velocity components) allows to model the energetic content of the subgrid-scale motions. The Reynolds fluxes that appear in PALM’s filtered equations (the spatial covariances of the subgrid-scale quantities) are parameterized by a flux-gradient approach, proportional to the resolved gradient and with a diffusivity coefficient that depends on the turbulent kinetic energy, the grid spacing and the height above the lower boundary of the simulation domain. At the first grid point above the surface, Monin-Obukhov similarity theory is applied to derive the horizontal velocity.

### LES set-up

We ran a suite of nine different cases with varying stability and a variable Bowen ratio, with periodic lateral boundary conditions and homogeneous surface conditions. The main parameters of these cases are summarized in Table 1. In practice, the surface fluxes and the geostrophic wind were set, together with the initial boundary-layer height (determined by the initial potential temperature profile). The remaining parameters and the final boundary-layer height are derived from the simulation results. In the coarse grid, the grid spacing is 25 m in the horizontal dimensions and 10 m in the vertical dimension. In the fine grid, the grid spacing is 5 m in the horizontal and 2 m in the vertical, leading to an isotropic nesting ratio of 5 between coarse and fine grid. In both fine and coarse grid the aspect ratio is 2.5. The domain size for the coarse grid is 5 x 5 km² in the horizontal, and 1.6 km in the vertical. The fine grid has the same horizontal extent but extends only up to 80 grid points (160 m). Due to the requirements of the nesting configuration, a fixed number of 25 cores was assigned to the coarse grid and 200 cores to the fine grid. In order to satisfy the matching constraints on the horizontal domain decomposition of the coarse grid and fine grid due to PALM’s MPI (Message Passing Interface) schemes, the coarse grid was split in 5 by 5 processor domains, and the fine grid in 10 by 20. The time step in all simulations was 0.25 seconds, allowing turbulent data output at 4 Hz. Each case was run for a total of 7 hours simulated time with the data extraction starting after 2 hours of spin-up time. The scalar and momentum advection schemes are Wicker-Skamarock, the pressure is solved with a Fast Fourier transform algorithm. The boundary conditions of the simulation are periodic in the lateral dimensions. For the velocity we have Dirichlet conditions at the bottom (i.e. rigid no-slip conditions) with zero vertical and horizontal wind. At the top of the domain the horizontal velocity is set to the geostrophic wind and the vertical velocity is zero. The geostrophic wind is along *x*, uniform with height and leads to a horizontal pressure gradient. The latitude is 38 degrees North. For the vertical velocity we have added a very small subsidence term (leading to a vertical pressure gradient in the equations) to counteract the destabilizing influence of the surface heat flux. The subsidence velocity at the surface is 0 m s^−1^ and the vertical subsidence velocity gradient is −4∙10^−5^ s^−1^ between 0 and 800 m and −2∙10^−5^ s^−1^ between 800 and 1000 m. For potential temperature and humidity we have Neumann conditions at the lower boundary (given by the surface fluxes) and also at the top boundary (given by the lapse rate at initialization). The domain is initialized with constant profiles for the velocity (equal to the geostrophic wind for x and y and zero for the vertical velocity). The initial profiles are homogeneous in x and y. For potential temperature (θ) the surface value is 300 K with a vertical gradient of 3∙10^−3^ K m^−1^ between 40 and 800 m and 8∙10^−3^ K m^−1^ above. For humidity the surface value is 1∙10^−3^ and the vertical gradient is zero, except between 1000 and 1100 meter, where it is −1∙10^−5^ m^−1^. The top of the domain is situated within a stable inversion layer, which prevents that the turbulence within the boundary layer is influenced by the vertical domain size. In the lateral dimensions the domain is about 5 times the boundary-layer depth. The surface roughness is *z*_*0*_ = 0.3 m.

**Table 1:** Parameters characterizing the simulated atmosphere for each simulation. The case names represent near-neutral (NN), weakly unstable (WU), moderately unstable (MU), strongly unstable (SU), and free convection (FC). *Inc* denotes the incoming energy (sum of the surface fluxes) and *z*_*i*_, u_*_ and w_*_ are time-averaged values during the data output.

	*U*_*g*_ (m s^−1^)	*H*_*s*_ (W m^−2^)	*λE*_*s*_ (W m^−2^)	*Inc* (W m^−2^)	*z*_*i*_ (m)	u_*_ (m s^−1^)	w_*_ (m s^−1^)	*Bo* (-)	*-z*_*i*_/*L* (-)
**NN1**	10	39.7	13.2	53	563	0.414	0.61	3.00	3
**WU**	10	65.5	23.5	89	555	0.444	0.72	2.78	4
**MU**	5	91.8	26.2	118	590	0.301	0.82	3.50	20
**MU2**	5	93.1	97.9	191	600	0.303	0.84	0.95	21
**MU1**	2	53.3	98.7	152	509	0.165	0.67	0.54	69
**SU**	2	196.7	60.3	257	784	0.194	1.16	3.26	213
**SU1**	1	106.6	197.4	304	642	0.124	0.92	0.54	407
**SU2**	2	198.5	196.5	395	806	0.194	1.19	1.01	230
**FC**	0.01	300.5	57.5	358	965	0.040	1.42	5.23	45909

The values in Table 1 for the cases WU through FC are based on Patton et al. [32] but not exactly identical, which can be explained by the absence of an explicit canopy in our simulations and the different surface model. Case NN1 has a considerably lower wind speed than their NN to allow for the same output frequency as the other simulations. The cases WU1, WU2, SU1 and SU2 are added to investigate the effect of a variable Bowen ratio.

### Data analysis

We employ two procedures to derive the turbulent fluxes. The first procedure is to calculate the fluxes from fully turbulent time series recorded at 10 x 10 virtual towers. The horizontal position of the towers corresponds to a 2D array with *x* = 500 *i* and *y* = 500 *j* with *i*,*j* = 1:10. The turbulent time series are recorded for 10 heights levels between 5 and 25 meters (with the fine grid vertical resolution of 2 m). We will use these data for the analysis of the local variability. The other procedure is to calculate the 3D time averages of fluxes online, such that only the time-averages and covariances are output, which allows the analysis of 3D information without the need to store 4D turbulent arrays. From both procedures, we obtain a local temporal flux (i.e. the Reynolds flux) by $\overset{-}{w\prime \theta \prime }$. The overbar denotes time-averaging, which is defined here as a block average over a certain half hour time window. The single primes denote the temporal (and local) fluctuation with respect to the local time mean $w^{\prime }(x,y,z,t)=w(x,y,z,t)-\overset{-}{w}(x,y,z)$. The Reynolds flux derived from the temporal covariance has to be distinguished from the spatial covariance obtained by horizontal averaging of the spatial fluctuations $\langle \overset{-}{w“\theta “}\rangle$. The angular brackets denote the horizontal averaging over all *x* and *y* grid points for a certain height level *z* and for a certain half hour time interval *w*"(*x*, *y*, *z*, *t*) = *w*(*x*, *y*, *z*, *t*) − 〈*w*〉(*z*, *t*). The double primes denote the spatial fluctuation with respect to the horizontal mean. The spatial covariance is only well-defined for an area average over the whole horizontal domain and it is related to the Reynolds flux by

$$\langle \overset{-}{w“\theta “}\rangle =\langle \overset{-}{w}\overset{-}{\theta }\rangle +\langle \overset{-}{w\prime \theta \prime }\rangle$$ (2)

We investigate the residual for the latent and sensible heat flux separately, and the total residual is found by the sum of both when expressed in energy units. We define the local sensible heat flux balance ratio as the Reynolds flux divided by the surface flux. The latter is prescribed in the model as a boundary condition. The local imbalance ratio (*I*) is then the flux balance ratio subtracted from one, e.g. for sensible heat:

$$I_{H}=1-\frac{\overset{-}{w\prime \theta \prime }}{H_{s}}$$ (3)

For the local imbalance, no spatial averaging is performed. For the normalization of the measured fluxes (temporal covariance), we divide by the surface flux in Eq 3, as we are primarily interested in comparing the measured flux to the available surface flux. Several previous studies [15,26] divide the temporal covariance by the spatial covariance because of their interest in the lower mixed layer where the storage term is larger- by normalizing with the spatial covariance the storage term does not influence the computed imbalance ratio anymore. For latent heat, *I*_*E*_ is found by replacing the potential temperature by the specific humidity *q* (and *H* is replaced by *λE*). The energy balance ratio (*EBR*) is not simply the sum of the flux balance ratios because of the different denominators, but instead

$$EBR=\rho \frac{c_{p}\overset{-}{w\prime \theta \prime }+\lambda \overset{-}{w\prime q\prime }}{H_{s}+{\lambda E}_{s}}$$ (4)

where *ρ* represent the air density, *c*_*p*_ the specific heat capacity of the air and *λ* the specific heat of evaporation. In Eq 6, we will explicitly consider the Bowen ratio and the stability index as parameters in the functional dependence of *I*_*H*_, in addition to the local variation given by *x*, *y*, *z*. Bowen ratio and stability index are also used to discriminate between the different simulations. The simulation setup is in practice determined by surface fluxes (*λE*_*s*_ and *H*_*s*_) and the geostrophic wind, but we combined these three parameters into dimensionless quantities, with *Bo* = *H*_*s*_/*λE*_*s*_. The stability index is derived for the whole domain, i.e. we take the average u_*_ and average w_*_:

u*w*=-(ziκL)-1/3 (5)

Note that there is also an implicit time dependence on the averaging interval in (3), and we define *I* to be positive for an underclosure, which is commonly the case.

Inspired by the parameterization of Huang et al. [26], we apply the following factorization for the local imbalance, for sensible and latent heat separately:

$$I_{H}(x,y,z;Bo,\frac{u_{*}}{w_{*}})=F_{1H}(\frac{u_{*}}{w_{*}})F_{2H}(\frac{z}{z_{i}})F_{3H}$$ (6)

Here, *F*_1*H*_ and *F*_2*H*_ are scaling functions for the sensible heat flux, different from those for latent heat, with *F*_3*H*_.capturing the remaining variability (with a different *F*_3*E*_ for latent heat as well). This decomposition implies that we assume that the vertical behavior of the flux imbalance can be described by a single function independent of the horizontal position and that the average flux imbalance only depends on the stability. Since we are studying homogeneous surfaces and the external parameters of our simulations can be grouped by the dimensionless numbers *Bo* and u_*_/w_*_, the average flux imbalance should only depend on these two. Furthermore, in field measurements there is also a relationship between stability and measured non-closure [8]. The assumption on *F*_*1H*_ can be reworded that the energy partitioning at the surface (as long as it leads the same stability index) does not influence the flux imbalance. When the assumption on *F*_*1H*_ holds, it follows that for a homogeneous simulation the additional vertical dependence in the (horizontally) mean flux imbalance should not depend on the horizontal position, and has to be relative with respect to the boundary-layer height.

To derive the scaling functions from our simulations, we proceed as follows: *F*_1_ captures the dependence on the stability of the atmospheric boundary layer. It is obtained by the horizontal means of *I* at a height *z* = 0.04 *z*_*i*_ in the surface layer, while Huang et al. [26] determined this function for the lower mixed layer between 0.3 and 0.5 *z*_*i*_. By fitting $\langle I_{H}(x,y,0.04z_{i};Bo,\frac{u_{*}}{w_{*}})\rangle$ we obtain *F*_1*H*_ = *a* exp(b u_*_/w_*_) + *c*. *F*_2_ captures the remaining height variation and is derived by fitting 〈*I*_*H*_ (*x*, *y*, *z*)〉/*F*_1*H*_(u_*_/w_*_) to *F*_2*H*_ = *q* + *m z*/*z*_*i*_, and similarly for the latent heat. *F*_3_ expresses the remaining variation due to the horizontal position and it also captures any deviation from u_*_/w_*_ and *z*/*z*_*i*_ scaling. For *F*_3_ we will consider the influence of (local) turbulence characteristics such as the streamwise-vertical velocity correlation coefficient $R_{uw}=\overset{-}{u\prime w\prime }/\left(\sqrt{\overset{-}{{u\prime }^{2}}}\sqrt{\overset{-}{{w\prime }^{2}}}\right)$, the friction velocity $u_{*}=\sqrt[{4}]{{\overset{-}{u\prime w\prime }}^{2}+{\overset{-}{v\prime w\prime }}^{2}}$ derived from the local momentum flux, the kurtosis $\overset{-}{{w\prime }^{4}}$ and skewness $\overset{-}{{w\prime }^{3}}$. These turbulence characteristics are derived for each tower location differently, as we want to relate the local remaining imbalance to the local turbulence.

### Statistical model

Using the 100 virtual towers from the LES time series output, we compute the correlations between the reduced flux balance ratios for sensible heat and latent heat, i.e.

$$I_{H}^{r}(x,y,z)=\frac{I_{H}}{F_{1H}(u_{*}/w_{*})F_{2H}(z/z_{i})},$$ (7)

$$I_{E}^{r}(x,y,z)=\frac{I_{E}}{F_{1E}(u_{*}/w_{*})F_{2E}(z/z_{i})},$$ (8)

and local turbulence variables, including *R*_*uw*_, u_*_, skewness and kurtosis of vertical velocity, the difference between the potential temperature and surface temperature (hereafter Δ*T*), the difference between the humidity and surface humidity (hereafter Δ*q*), and triple variances $\overset{-}{w^{\prime }w^{\prime }T^{\prime }}$ and $\overset{-}{w^{\prime }w^{\prime }q^{\prime }}$. We denote the correlation coefficient computed from these virtual tower measurements as *R*_*m*_, e.g. $R_{m}(I_{H}^{r},\overset{-}{w^{\prime 3}})$ for the correlation between the reduced sensible heat flux balance ratio and the vertical velocity skewness, to distinguish it from e.g. the correlation coefficient derived from spatial data *R*_*S*_.

Aiming at building a linear model out of the above variables that contribute to $I_{E}^{r}$ (resp.$I_{H}^{r}$), several statistical methods are employed. First, outliers of the predicting data are identified by the Tukey rules [39] and then replaced with k-nearest neighbor imputation method [40]. Second, we select significant variables by checking for *p*-values and compute the relative importance of variables [41]. Then the variables have been filtered and sorted as the most suited variables to explain the imbalance ratio [42]. By using stepwise model selection, all combinations of selected variables are subjected to information criterion analyses to identify the “best” model, that is, a model with lower Akaike information criterion (AIC) value [43] and higher coefficient of determination *R*^2^ and adjusted *R*^2^. This regression procedure was applied to all the virtual tower measurements from 10 m to 26 m for all the cases.

## Results

### General characteristics of the simulations

**Fig 1:**
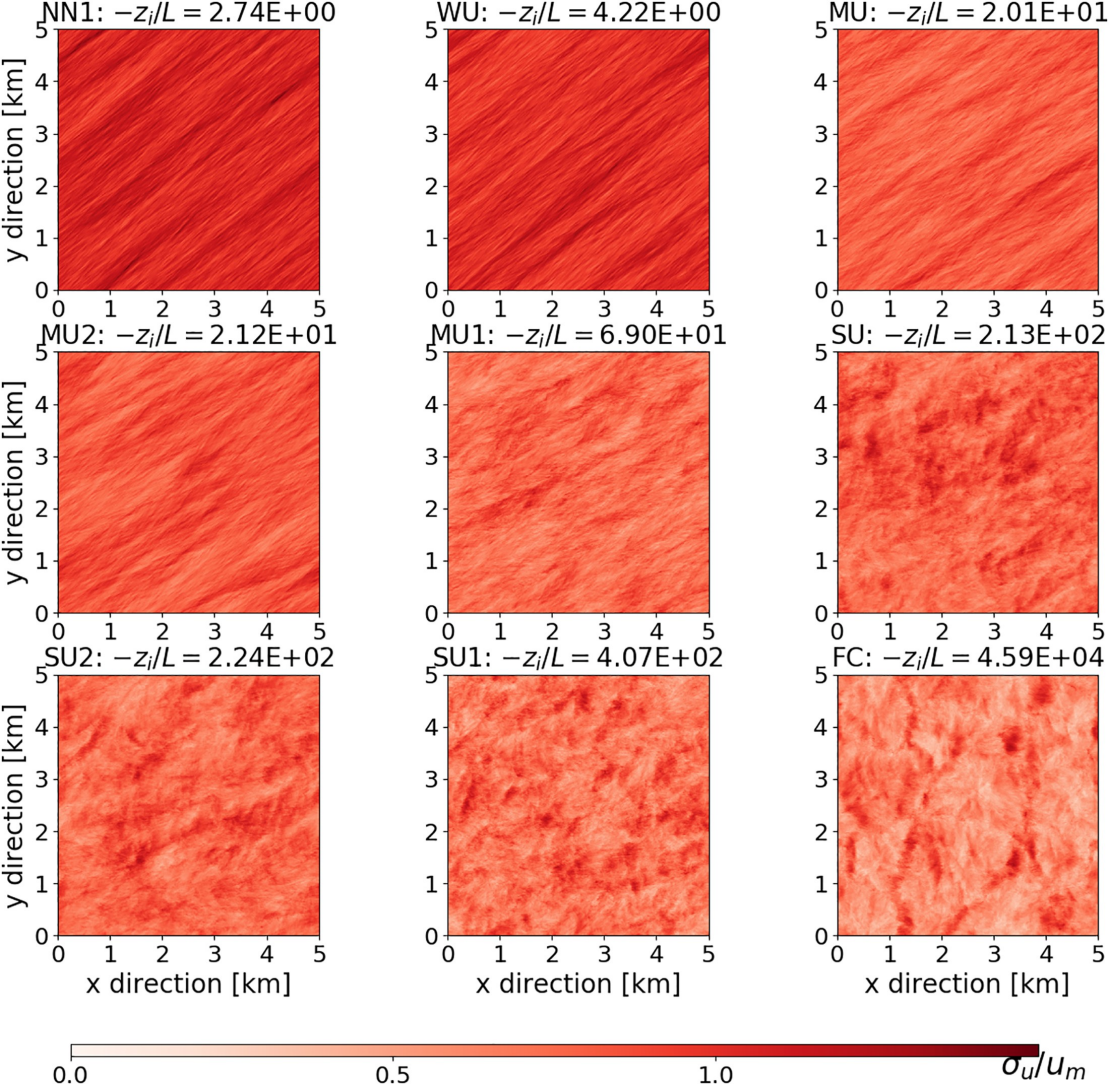
Standard deviation of streamwise velocity (*σ*_*u*_) at 20 m height as a function of stability, and normalized by $u_{m}=\sqrt[{3}]{5{\left(u_{*}\right)}^{3}+{\left(w_{*}\right)}^{3}}$ [44].

Before we turn to the dependence of the imbalance on stability and height, we discuss the general behavior of the different cases. In Fig 1, we show horizontal cross-sections for the standard deviation on the streamwise velocity at 20 m height in the fine grid. In the simulations the geostrophic wind is prescribed, which implies that the surface wind has a different direction for different stability, with a more pronounced Ekman spiral for the less unstable cases. With rising instability, the structures change from elongated streaks over rolls to rotationally invariant structures corresponding to the typical hexagonal structures that appear in the vertical velocity for convective conditions.

**Fig 2:**
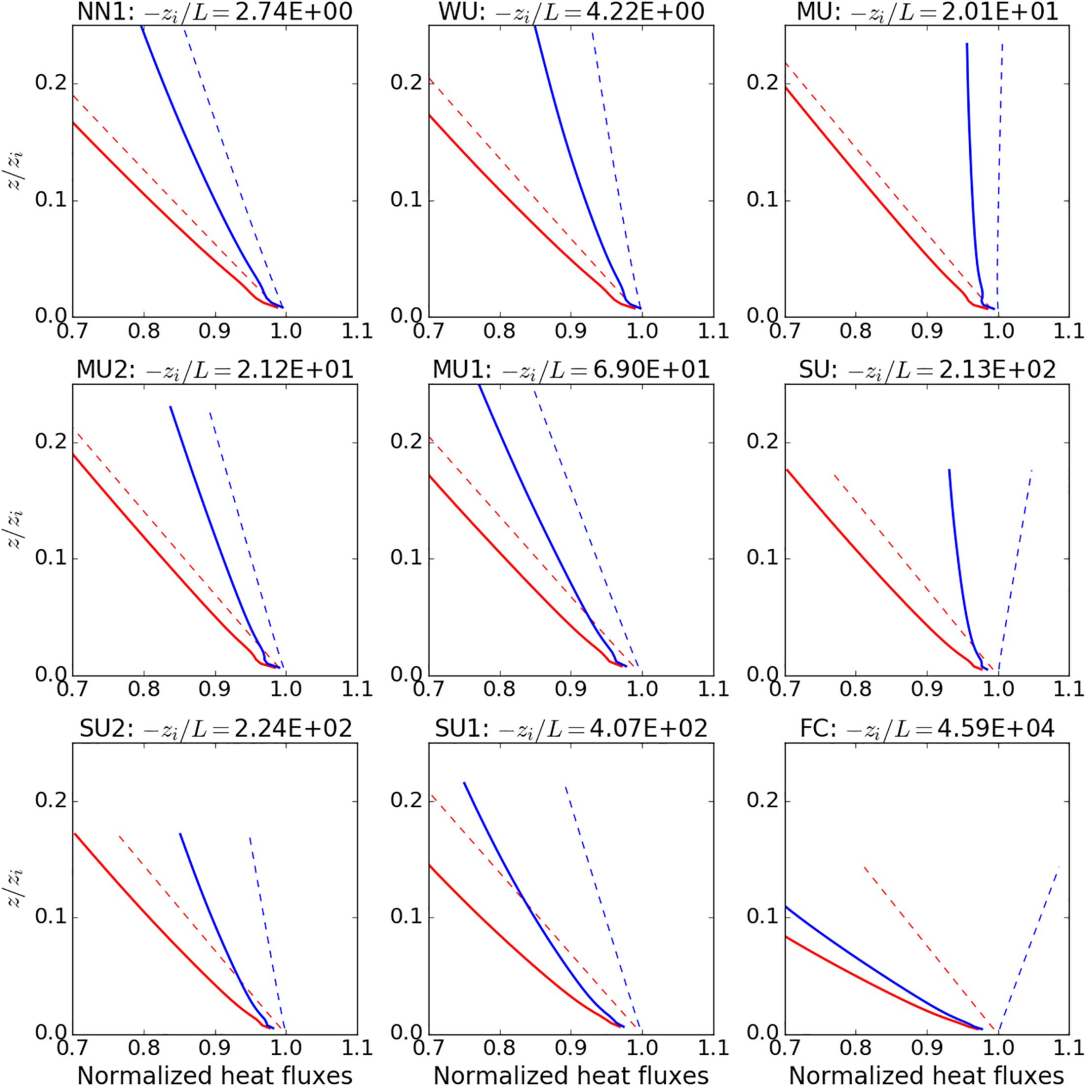
Area-averaged spatial covariances (dashed lines) and temporal covariances (solid lines) for the different stabilities, with data up to 160 m from the fine grid, and normalized by the surface fluxes. Red curves are for the sensible heat fluxes and blue curves for the latent heat fluxes.

As can be seen from the vertical profiles for the different cases (Fig 2), the fluxes vary linearly with height in the surface layer. The latent heat does not necessarily always decrease with height in the surface later. Especially the spatial covariance of the latent hear for the cases with high instability and high Bowen ratio, shows an increase in the surface layer. These profiles show the advantage of our nesting method, which allows to resolve the turbulence also for small *z*. Temporal virtual measurements (not shown) from our simulations also reproduce the temporal saw-blade pattern (ramp-like structures) from rapid changes in the turbulent heat fluxes, which have been described in several experimental studies [14,35,45,46]

### Horizontal and vertical distribution of the imbalance for one particular case

**Fig 3:**
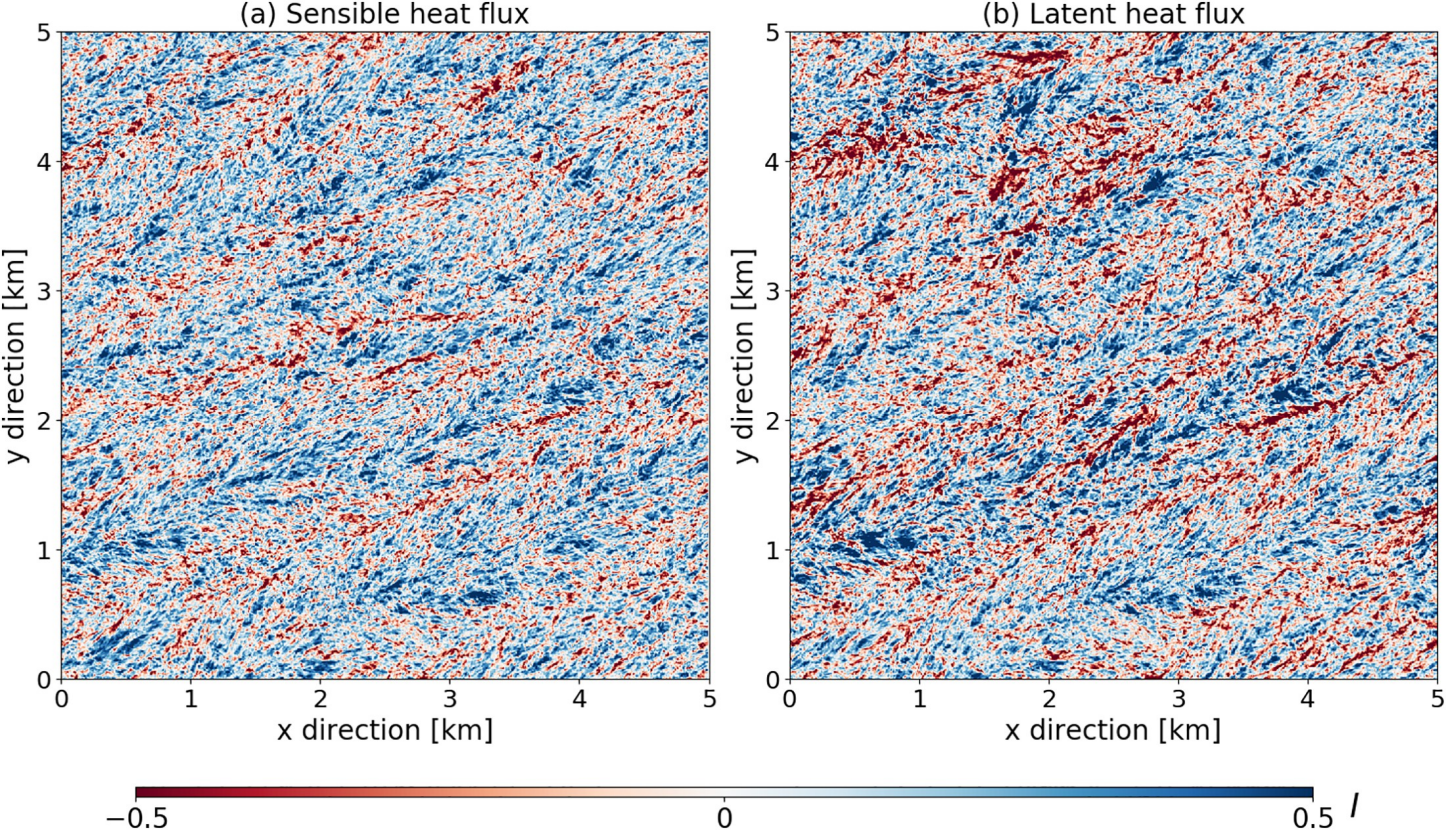
Horizontal cross sections for the SU case, at 20 m height, for the last hour of time-averaged data. The imbalance term is derived for the sensible and latent heat fluxes separately. Blue is underclosure (positive *I*), red is overclosure (negative *I*).

**Fig 4:**
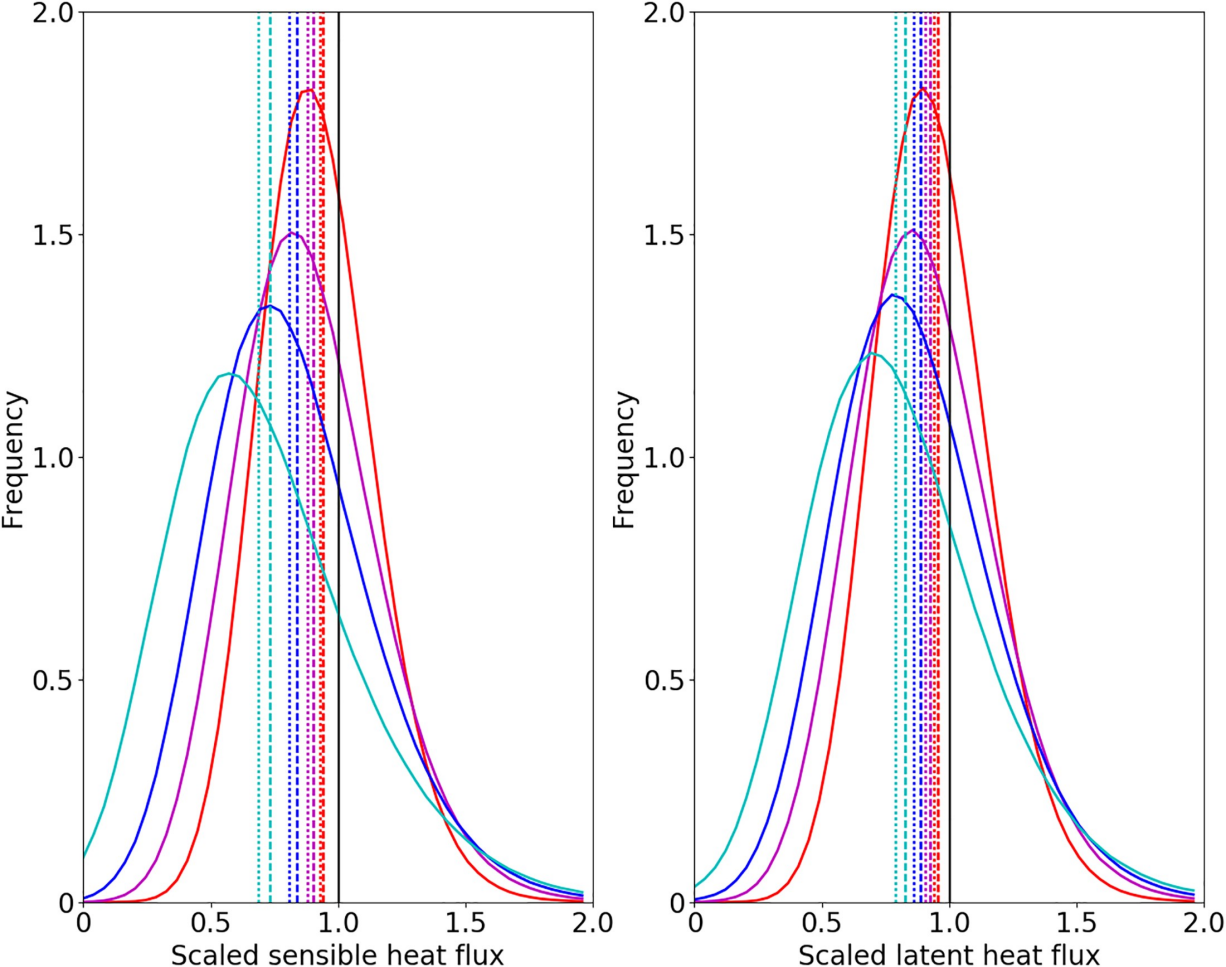
Probability density functions showing the horizontal variability of the Reynolds flux scaled by the surface flux at different height levels. Plotted data are for the SU case, with the left panel the sensible and the right panel the latent heat flux. The heights are at 10 m (red), 20 m (magenta), 40 m (blue) and 80 m (cyan). The dashed lines represent the arithmetic mean and dashed-dotted lines the median.

We take a closer look at the spatial variation of the imbalance ratio for the strongly unstable case (Fig 3). As we will study in more detail in Fig 4, the blue regions with underclosure are more common than the red regions with overclosure. Interesting is the different location of pronounced *I*_*E*_ and *I*_*H*_ structures, a maximum in *I*_*H*_ does not correspond to a maximum in *I*_*E*_. However, the location of the blue and red regions appears to correspond quite well on average, even when the magnitude for the structures in *H* and *λE* differs. We can check this more quantitatively by computing the spatial correlation coefficient between the temporal covariance for the potential temperature and humidity fluxes given by

$$R_{s}(\overset{-}{w^{\prime }\theta^{\prime }},\overset{-}{w^{\prime }q^{\prime }})=<(\overset{-}{w^{\prime }\theta^{\prime }})“(\overset{-}{\text{w}\prime \text{q}\prime })“>/\left(\sqrt{{<(\overset{-}{\text{w}\prime \theta \prime })“}^{2}>}\sqrt{{<(\overset{-}{\text{w}\prime \text{q}\prime })“}^{2}>}\right)$$ (9)

This coefficient has values ranging between 0.7–0.9 for the different stabilities, which shows that the imbalances for the fluxes exhibit horizontal patterns that are correlated. A similar computation for $R_{s}(\overset{-}{w^{\prime }\theta^{\prime }},\overset{-}{w})$ and $R_{s}(\overset{-}{w},\overset{-}{w^{\prime }q^{\prime }})$ yields lower values, between 0.4 and 0.6 for the different cases, with the slightly higher values for less unstable conditions. This shows that the overclosure is moderately linked to updrafts and the underclosure to downdrafts, but with more significant scatter than the linking between the positions of the structures for the latent and sensible heat flux.

To investigate the spatial variation of the imbalance ratios in more detail, we computed probability density functions for the horizontal variability of the flux balance ratio at different height levels (Fig 4). The dashed lines indicate the mean of the distribution, the dashed-dotted lines the median of the distribution. This shows that all the pdfs are skewed, with the tail to the right, but the mean and median lie below 1 in every case. As was already slightly apparent in Fig 3, the underclosure dominates the distribution but the tail to the right means that there are also positions with large overclosure and there is significant horizontal variation.

### Dependence on atmospheric stability

**Fig 5:**
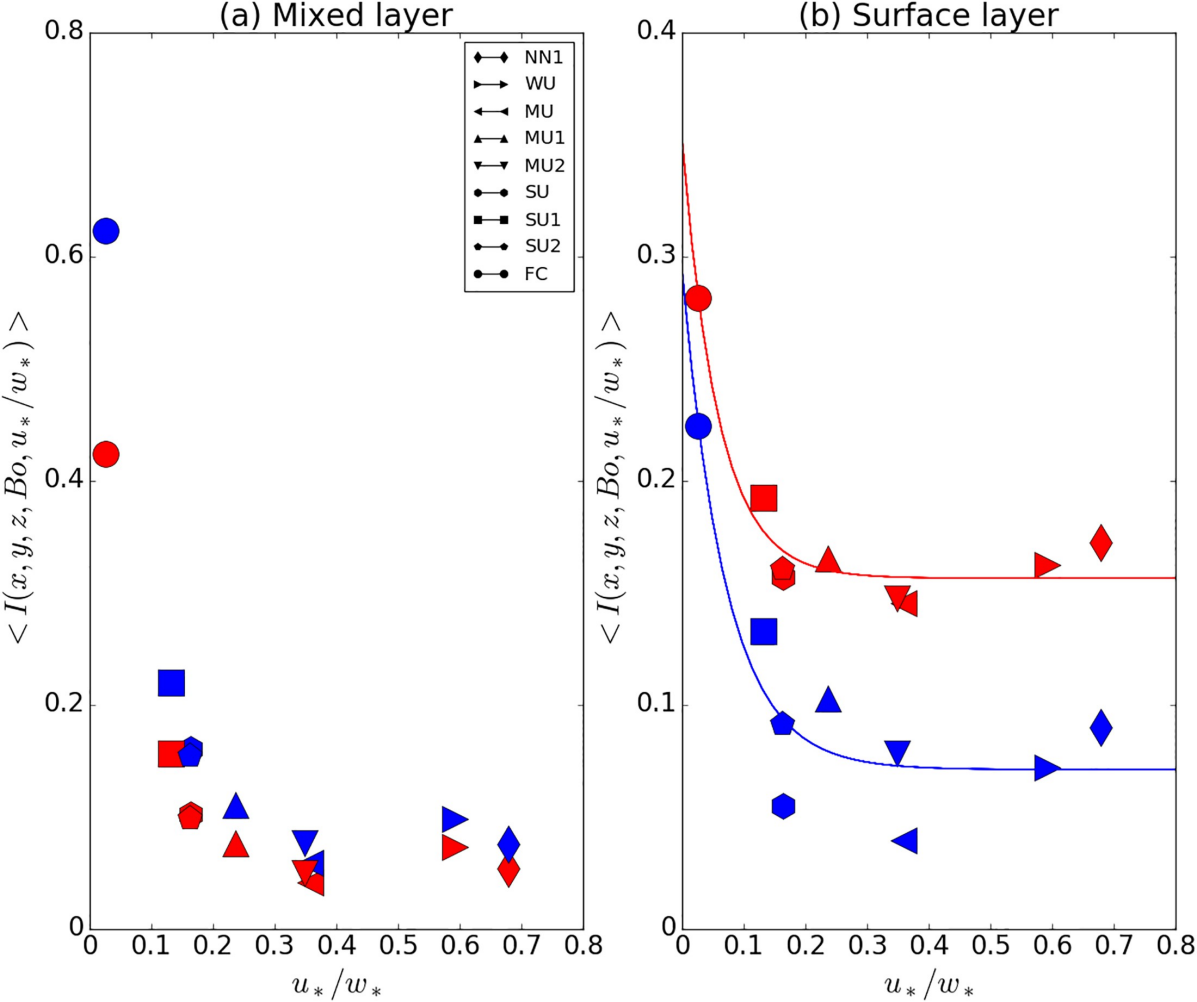
Imbalance ratios in the mixed layer (a) and the surface layer (b), as a function of the stability parameter u_*_/w_*_. Please note the different range of the ordinates in the panels, because the imbalance is larger in the mixed layer than in the surface layer. The surface layer imbalance was computed at *z* = 0.04 *z*_*i*_, while the mixed layer imbalance was calculated from a vertical average between [0.3 *z*_*i*_ − 0.5 *z*_*i*_]. Red markers are used for the sensible heat data (normalized potential temperature flux) and blue markers for the latent heat data (normalized humidity flux). It is important to note that the data in panel (a) are normalized with the spatial covariance. In the surface layer, the normalization is with the surface flux. The fits for *F*_1H_ and *F*_1E_ are given by the red and blue curves, respectively. We find as the coefficient of determination *R*^*2*^ = 93% for *F*_*1H*_ and *R*^*2*^ = 82% for *F*_*1E*_. For the standard error of the regression in natural units we find *S* = 0.30 for *F*_*1H*_ and S = 0.48 for *F*_*1E*_.

As the first step in the quantification of *I*_*E*_ and *I*_*E*_ in the surface layer, we investigate the stability dependence of the area-averaged flux imbalance. The panel of Fig 5A shows results in the mixed layer for comparison with Huang et al. [26]. The agreement with the earlier work is very good, which shows, that the simulation results are robust and that the choice of the LES model does not introduce artifacts. In Fig 5B the results for the surface layer, < *I*_*H*_ (*z* = 0.04 *z*_*i*_) > and < *I*_*E*_ (*z* = 0.04 *z*_*i*_) >, are shown. It is interesting to note that in the mixed layer the imbalance ratio for the latent heat flux lies above that for the sensible heat flux but that for the surface layer the imbalance ratio for the latent heat flux lies below that for the sensible heat flux.

As we are interested in fitting the stability dependence in the surface layer and the relation of the fluxes with respect to the true surface fluxes, we derive the following fits for the normalized latent and sensible heat imbalance ratios presented in Fig 5B:

$$F_{1H}=0.197\;\text{exp}(-17.0\;u_{*}/w_{*})+0.156$$ (10)

$$F_{1E}=0.224\;\text{exp}(-14.0\;u_{*}/w_{*})+0.071$$ (11)

For the latent heat flux, the spread around the *F*_1*E*_ fit is considerably larger than for the sensible heat flux. This can probably be related to the variable Bowen ratios, which suggests that the first fitting function for the latent heat flux would need an additional dependence on the Bowen ratio, and not just on the stability, i.e. *I*_*E*_ (u_*_/w_*_, *Bo*). However, with three SU cases and three MU cases, we do not have enough data to derive a two-dimensional fit.

On the other hand, it is intriguing that the *F*_1*H*_ fit works well without a dependence on the Bowen ratio. This probably implies that a lower Bowen ratio (from a higher surface latent heat flux) does influence the energy balance closure for latent heat, but not inasmuch for sensible heat. However, if we suppose a mechanism where the Bowen ratio influences the EBR through the buoyancy flux and the resulting convective turbulence, it should have an effect on both fluxes, because they are affected by the same turbulent structures. We therefore check the hypothesis that the influence of Bowen ratio, disturbing pure u_*_/w_*_ scaling in *F*_1*E*_, might actually appear from entrainment effects. For this purpose we computed the ratio between the entrainment flux and the surface flux, for each case and for both fluxes. Aside from the FC case, which has much larger ratio of the entrainment flux with respect to the surface flux due to its more vigorous convection and higher boundary-layer height, the ratios for the other cases do not vary much for sensible heat, which would be consistent with the relatively clean u_*_/w_*_ scaling in *F*_*1H*_. The latent heat flux however shows some variation, where, the cases with lower Bowen ratio exhibit smaller ratios too, by up to one order of magnitude. The simulations with lower Bowen ratio (SU2, MU1, MU2) lie above the *F*_1*E*_ fit, i.e. they have higher imbalance than their drier counterparts. In order to investigate this issue further, a larger range of Bowen ratios should be explored, perhaps in addition with purely passive bottom-up and top-down tracers, in order to understand if the buoyancy flux does play a role, and to what extent entrainment is involved.

### The influence of measurement height on the imbalance

**Fig 6:**
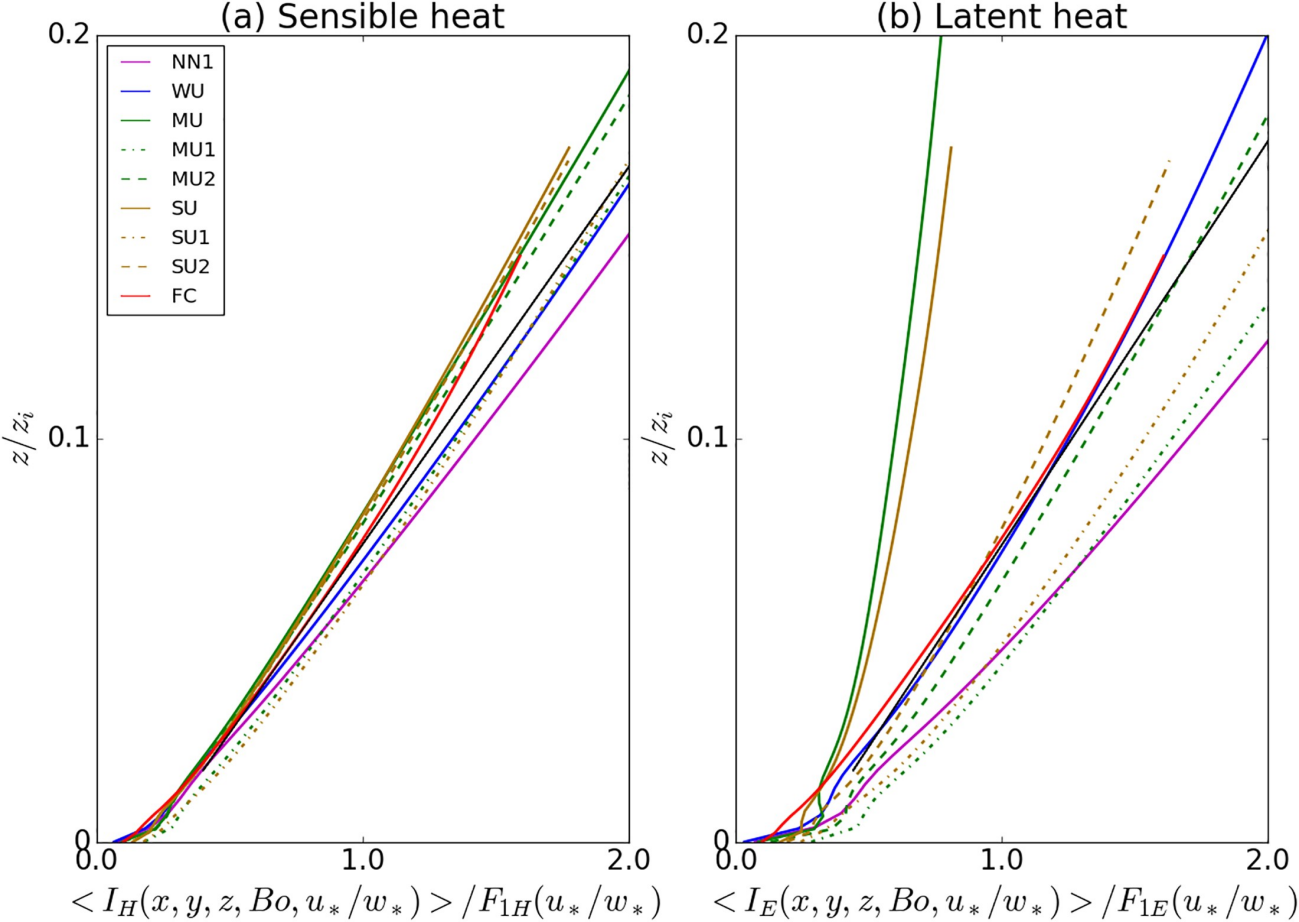
Vertical dependence of the horizontally averaged imbalance ratio for different stabilities divided by the stability fit, with vertical values up to 160 m. The case-independent fit is shown by the black line. Sensible heat flux in panel (a), latent heat flux in panel (b).

After division by the stability dependence, the next step is to fit the vertical dependence of the remaining imbalance. For higher measurement heights the imbalance generally becomes larger due to the advection and storage terms, which is ultimately a consequence of the wind speed growing with height, and the larger air mass below the measurement position. The vertical variability of all the cases is shown in Fig 6. The fitting procedure leads to:

$$F_{2H}=0.21+10.69\;z/z_{i}$$ (12)

$$F_{2E}=0.27+9.99\;z/z_{i}$$ (13)

The divergence of the vertical profiles for latent heat in the different cases is due to the larger spread of the data points around *F*_1*E*_ (see Fig 5B) and is not a consequence from *I*_*E*_ itself. It is interesting to note that exactly the same cases where the data points lie above or below the fit in Fig 5B correspond in Fig 6B to the cases with profiles to left and right of the case-independent height fit. For the sensible heat flux, the vertical profiles collapse much better onto one case-independent vertical profile, which corresponds to the finding that for sensible heat *F*_1*H*_ can be approximated as independent of the Bowen ratio.

### The local influence on large eddy transport

To investigate the remaining local variability in the flux measurements, we consider the reduced flux imbalance at every point, where the stability and the height dependence have been divided out, e.g. for sensible heat (similar for latent heat):

$$I_{H}^{r}(x,y,z)=\frac{I_{H}}{F_{1H}(u_{*}/w_{*})F_{2H}(z/z_{i})}$$ (14)

**Fig 7:**
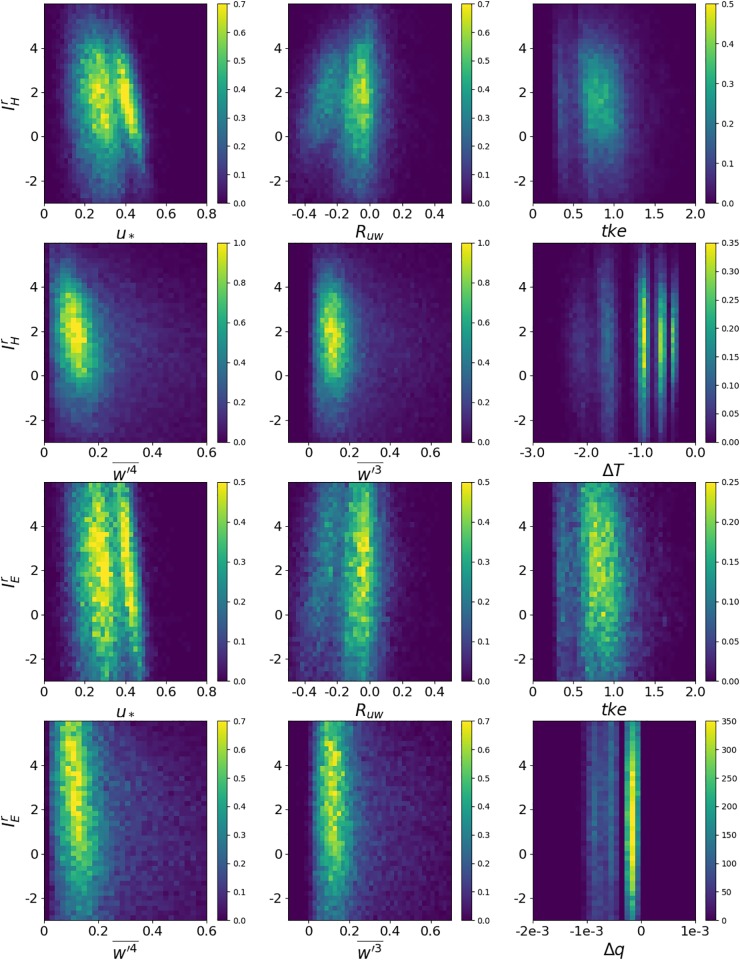
Two-dimensional joint-probability density plots for six variables with reduced imbalance ratio for sensible heat and latent heat, respectively; i.e. the density plots of < *I*(*x*, *y*, *z*) >/(*F*_1_ (u_*_⁄w_*_)*F*_2_ (*z*⁄*z*_*i*_)) in function of 6 variables u_*_, *R*_*uw*_, tke, $\overset{-}{w^{\prime 3}},\overset{-}{w^{\prime 4}}$, and Δ*T* or Δ*q* with the latter being the temperature (moisture) difference between the measurement point and the surface. The respective variables are listed at the abscissa. The different scales for the density follow from the range of the variables, due to the normalization condition on the probability density. For the density plots half-hourly averaged data from all 10 time intervals were considered.

In Fig 7, density plots for the reduced imbalance for sensible and latent heat and six selected turbulence characteristics are shown, which are constructed from 100 virtual towers, at six vertical levels between 13 m and 25 m, for ten half hour measurements, with all cases combined. We note that there are two lobes for the joint distribution functions with u_*_ and *R*_*uw*_. This suggests that the data could be separated with respect to another variable. However, the multiple lobes for the temperature and humidity differences clearly originate from the different stability indices, due to our discrete number of cases. For the temperature and moisture difference we only consider the difference between the measurement height and the surface, without dividing by the measurement height as would be done for the gradient. We do so because the main temperature difference is in the superadiabatic layer immediately above the surface and dividing by the height would therefore decrease the correlation. We note again that the latent heat imbalance has more spread, but this is due to the *F*_1*E*_ fit which does not include a dependence on the Bowen ratio.

### Regression analysis for the reduced flux imbalance

In the final section we attempt to derive a statistical model for the reduced flux imbalance, based on local turbulence statistics. Large-scale turbulent structures were suspected to be responsible for the saw-blade pattern of the energy balance residual [14]. The authors of that study also found evidence that, if the background wind is high enough, there is strong mechanical mixing in the surface layer and the majority of the energy-transporting eddies are captured by the EC measurement. For this reason, the energy balance residual in their study is negatively correlated with the friction velocity or rather *R*_*uw*_, the latter varying considerably, ranging from −0.43 to −0.02 [47]. This dependence is well known and suggests that a high intensity of mechanically induced turbulence improves the energy balance closure [8,45,48–51].

**Fig 8:**
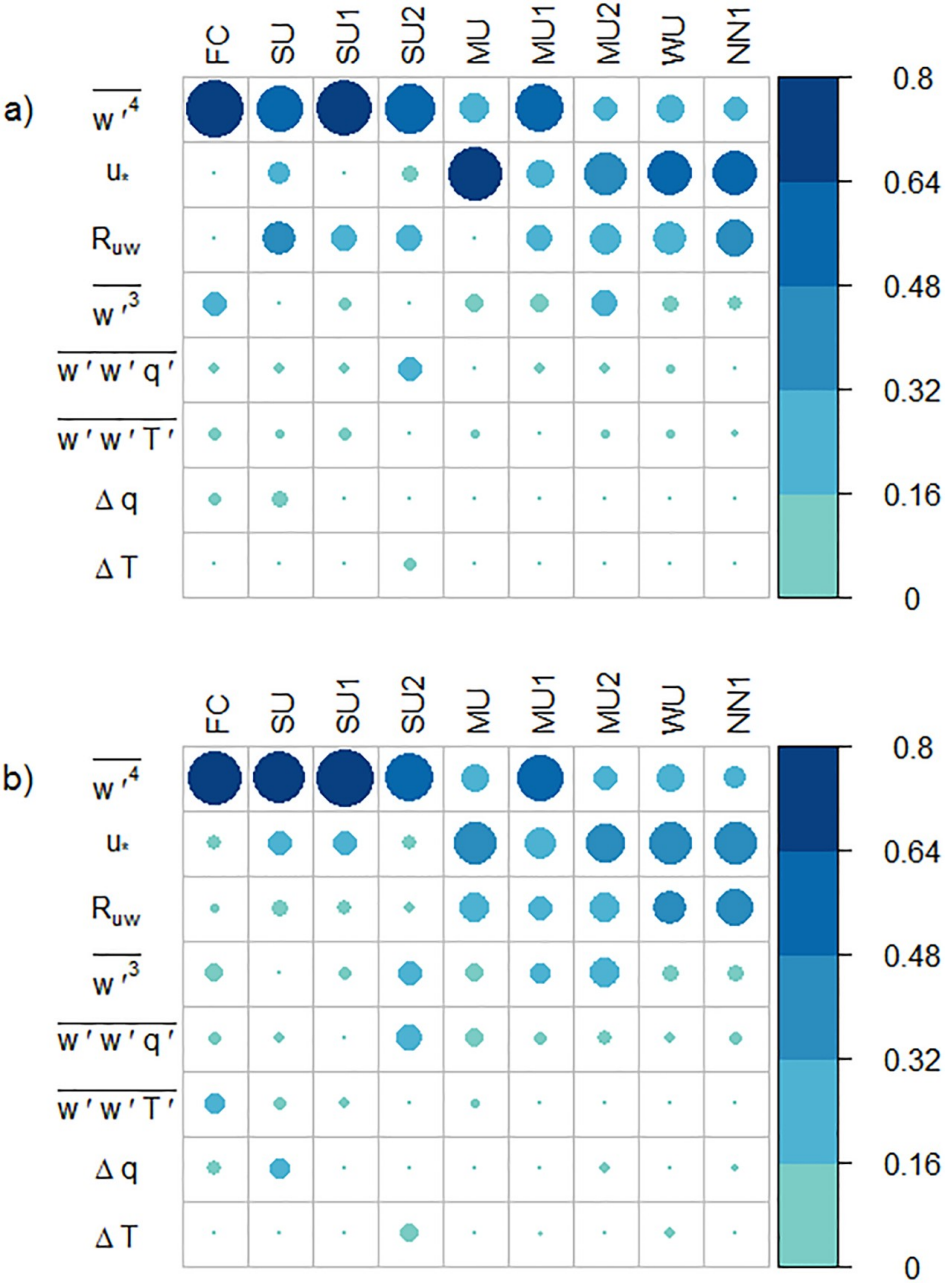
Relative importance of factors in the regression model for the latent heat flux (a) and the sensible heat flux (b).

The final step is to derive a regression model to explain the reduce energy imbalance. To this end, we computed the correlations between the reduced flux imbalance and the most significant turbulence variables, where the correlation is based on the virtual tower measurements as described in section 2.3. As the correlation result indicates, $I_{E}^{r}(I_{H}^{r})$ is significantly related to u_*_, *R*_*uw*_, $\overset{-}{w^{\prime 3}},\overset{-}{w^{\prime 4}}$, Δ*T*, Δ*q*, $\overset{-}{w^{\prime }w^{\prime }T^{\prime }}$ and $\overset{-}{w^{\prime }w^{\prime }q^{\prime }}$. Fig 8 illustrates the relative importance of variables in the fitted model. Obviously, some of these variables are also correlated among each other, but our aim is to find out which correlate better with the reduced flux imbalance. Similar to previous studies [8,20,52], we see significant correlation between $I_{E}^{r}(I_{H}^{r})$ and u_*_. $R_{m}(I_{H}^{r},R_{uw})$ is negative in all the cases, which agrees with the results of Zhang et al. [35] and Eder et al. [52] that half-hourly *R*_*uw*_ variations correspond to variations of the latent heat flux. Furthermore, for both sensible and latent heat, the absolute values of correlation coefficients tend to increase with height and stability, especially for $R_{m}(I_{H}^{r},R_{uw})$ and $R_{m}(I_{H}^{r},u_{*})$ (height dependence is not shown).

The results for the latent heat flux and the sensible heat flux are similar: in the cases FC, SU1, SU2, MU, $\overset{-}{w^{\prime 4}}$ ranks at the top in the contribution to the fitted model, while in the cases MU, MU2, WU and NN1, u_*_ contributes most strongly to the flux imbalance. The lower explanatory power of the statistical model for the case of free convection can be explained by the Taylor hypothesis underlying EC measurements, which is violated when the background wind becomes negligible. For sensible heat, *R*_*uw*_ follows a similar pattern as the friction velocity, albeit with secondary importance. For latent heat *R*_*uw*_ is also contributing to the more unstable cases. $\overset{-}{w^{\prime 3}},\overset{-}{w^{\prime }w^{\prime }T^{\prime }}$ and $\overset{-}{w^{\prime }w^{\prime }q^{\prime }}$ have a small influence in the model. Lastly, the contributions of Δ*T* and Δ*q* are the lowest in the fitted model, but this can probably be related to our set-up with homogeneous terrain, as these differences are expected to contribute mainly in heterogeneous terrain [20,53]. The difference between latent and sensible heat is not pronounced, with the exception of *R*_*uw*_.

Since our final goal is to derive a universal model for $I_{E}^{r}(I_{H}^{r})$, we applied the regression analysis with the whole dataset of all the cases on the level 20 m. However, both *R*^2^ and the adjusted *R*^2^ are relatively low, around 0.026, which means under the condition with all the stabilities, the fitted model cannot explain the reduced imbalance ratio well without separating the data by stability.

## Discussion

In the surface layer, we find a different parameterization than Huang et al. [26] found for the mixed layer. First of all, the imbalance ratio is naturally lower near the surface. It is however possible to retain the same functional shape for the fit in the surface layer as in the mixed layer. Remarkably, in the surface layer, the latent heat flux has lower imbalance ratios than the sensible heat flux, in contrast to the mixed layer. This larger underestimation of the sensible heat flux is in accordance with the findings with several other studies [54–56]. The dependence of the latent heat imbalance ratio on the Bowen ratio appears to be constricted to the surface layer, because our simulation data in the mixed layer for the same number of cases follows the results of Huang et al. [26] with no pronounced dependence on the Bowen ratio. To connect the fit in the surface layer with that in the mixed layer, the individual coefficients for *F*_1_ should be made height-dependent (as a function of *z/z*_*i*_), instead of a complete separation of the height dependence in *F*_*2*_. In addition, the storage flux should be taken into account in the mixed layer as well, by normalizing with respect to the surface flux instead of the spatial covariance at that height.

For a particular measurement interval we thus derive the following formulae for the residual sensible heat flux and the total sensible heat flux (with *H*_*m*_ being the measured sensible heat flux), under homogeneous conditions:

$$H_{res}=\frac{F_{1H}(u_{*}/w_{*})F_{2H}(z/z_{i})}{1-F_{1H}(u_{*}/w_{*})F_{2H}(z/z_{i})}H_{m}$$ (15)

$$H_{tot}=\frac{1}{1-F_{1H}(u_{*}/w_{*})F_{2H}(z/z_{i})}H_{m}$$ (16)

The same formulae (with *E* traded for *H*) apply for the latent heat flux. However, for the latent heat flux the correction factor has to be applied with more restraint, because the fit with respect stability works considerably less good than for sensible heat. For a scalar such as water vapor, entrainment at the top of the boundary layer affects the scalar transport, which may explain why the latent heat flux imbalance is higher than the sensible flux imbalance in the mixed layer, but it is unclear to what extent this may affect the behavior of the fluxes in the surface layer. We also stress that these formulae (15–16) do not allow the partitioning of the residual into a storage flux, flux-divergence or advection by the mean flow, it only quantifies the total residual.

The fact that unstable conditions lead to a larger flux imbalance, aligns well with the findings of Li et al. [57] who recently showed that the temperature similarity function conditioned on downdrafts does not follow Monin-Obukhov similarity scaling, in contrast to the similarity function conditioned on updrafts. For unstable conditions, the downdrafts are stronger and cover a larger area. The latent and the sensible heat flux behave similarly when deriving a statistical model between the reduced flux imbalance ratio and local turbulence variables. When separating the different stabilities, the major contribution for the less unstable cases comes from the friction velocity and the major contribution to the unstable cases comes from the kurtosis of the vertical velocity. An important question is whether this parameterization is limited to homogeneous surfaces. Due to the large spread in the probability distributions when considering the positional variation for homogeneous terrain (see Fig 4), we believe that our correction formulae might also hold in heterogeneous terrain on average, provided the turbulent structures are not pinned to a particular position, i.e. for small -*z*_*i*_/*L* such that the rolls effectively smear out the heterogeneity [23], and provided the heterogeneity is weak (i.e. the spread in the surface heterogeneity distribution is smaller than the spread of the distribution of the flux imbalance for the homogeneous case). However, the simulations of De Roo and Mauder [19] under free convection conditions and for distinct heterogeneity show that the flux underestimation of virtual eddy-covariance towers indeed depends on the location of the measurement with respect to the boundaries of a warm or cold patch under more unstable conditions or when the contrast in surface heating is large. This parameterization is therefore not applicable under such conditions. Moreover, the dependence of the flux imbalance on the measurement height is probably still underestimated near the surface due to the limited grid resolution of our LES, despite the vertical nesting. Therefore, this parameterization is limited to heights above 10 m. In addition, due to the limited range of Bowen ratios covered by our simulations, we suggest to apply these parameterizations only for Bowen ratios between 0.5 and 5.

Due to fluctuations in the half-hourly measurements and to suppress scatter [53], we propose to partition the residual in for weakly heterogeneous terrain based on the daily *EBR* (*EBR*_*d*_) by preserving the half-hourly ratios *H*_*res*_/*λE*_*res*_:

$$H_{tot}=H_{m}+\frac{H_{res}}{\left(H_{res}+{\lambda E}_{res}\right)}Res$$

$${\lambda E}_{tot}={\lambda E}_{m}+\frac{{\lambda E}_{res}}{\left(H_{res}+{\lambda E}_{res}\right)}Res$$

$$Res=(H_{m}+{\lambda E}_{m})(\frac{1}{{EBR}_{d}}-1)$$

As such, the scheme of Mauder et al. [58] can be followed, but with a different correction factor for latent and sensible heat. This correction assigns more residual to the sensible heat than the Bowen ratio preserving correction of Mauder et al. [58], but less than the correction suggested by Charuchittipan et al. [55]. This requires independent high-quality measurements of the energy fluxes and the best possible data for the height of the atmospheric boundary layer at the measurement site.

## Conclusions

We have derived a parameterization for the near-surface energy imbalance, and we have shed light on the processes and potential drivers of large-scale organized transport as a result of secondary circulations. This parameterization for correcting the eddy-covariance fluxes is particularly useful when not all other non-turbulent terms of the surface energy balance are not or at least not easily measurable with sufficient accuracy, e.g. for sites with tall vegetation canopy or very shallow soils, over lakes or in urban systems. Remarkably, we found that the sensible heat flux shows a larger underestimation in the surface layer than the latent heat flux, while the underestimation is very similar between both fluxes higher up in the mixed layer. Since eddy-covariance measurements are typically conducted in the surface layer, we conclude that a simple energy balance closure adjustment by conserving the Bowen ration cannot be supported. Therefore, we proposed different coefficients for the parameterizations of the spatially averaged sensible and latent heat flux imbalances. Their functional form is however identical, both depending on u_*_/w_*_ and *z/z*_*i*_. This means that the spatially averaged imbalance increases with increasing instability, and that the variability of the local imbalance also increases with increasing instability, both between different time intervals and between different towers, as these are just different sub-samples of the overall turbulence field. We also investigated the behavior of the remaining local imbalances and found that those depend on local variables, such as u_*_, *R*_*uw*_, and skewness and kurtosis of vertical velocity. However, no functional relationship was derived here for those additional variables because we considered their statistical correlations to be not strong enough unless they are classified by stability. A remaining question is the precise mechanism how the local turbulence characteristics influence the local energy balance. Perhaps one way to shed more light on this is how the variance in the spatial distribution for the imbalance ratio is related to the (averaged) turbulence characteristics. Finally, based on our simulation results, we propose a method to correct eddy-covariance measurements of the sensible and the latent heat flux.

## Supporting information

S1 Text Source code of the PALM model, including additional user-defined code, used for running the simulations for this study. (ZIP)

S2 Text Settings of the high-performance computing cluster where the results were produced. (ZIP)

S1 Dataset Parameter files with data set that specifies the model output. (ZIP)
